# Supplementary material for: Nitrates vs. Other Types of Vasodilators and Clinical Outcomes in Patients with Vasospastic Angina: A Propensity Score-Matched Analysis
Source: J Clin Med. 2022 Jun 7;11(12):3250. doi: 10.3390/jcm11123250 (PMC9225129; doi:10.3390/jcm11123250)
Supplement: Supplementary file 1 [file jcm-11-03250-s001.zip › jcm-1690450-supplementary.pdf]

SUPPLEMENTAL MATERIAL

Supplementary Table S1. Coronary artery spasm and associated characteristics after provocation test according to vasodilator

	All (n = 1048)	Nitrates (n = 239)	Other types of vasodilator (n = 809)	*p* value
Location of spasm				
Location of spasm (>2.5 mm vessel) LM	4 (0.4)	0 (0.0)	4 (0.5)	0.579
Location of spasm (>2.5 mm vessel) LAD	511 (48.9)	126 (52.7)	385 (47.8)	0.184
Location of spasm (>2.5 mm vessel) LCx	235 (22.5)	46 (19.2)	189 (23.5)	0.169
Location of spasm (>2.5 mm vessel) RCA	509 (48.8)	127 (53.1)	382 (47.5)	0.123
Associated ECG change	138 (13.2)	36 (15.1)	102 (12.6)	0.324
Associated chest pain	620 (59.2)	164 (68.6)	456 (56.4)	0.001
Associated arrhythmia†	149 (14.2)	28 (11.7)	121 (15.0)	0.207
Associated cardiac arrest	3 (0.3)	0 (0.0)	3 (0.4)	1.000

† Atrial fibrillation, atrial flutter, ventricular fibrillation, ventricular tachycardia, or atrioventricular block.

ECG, electrocardiography; LAD, left anterior descending coronary artery; LCx, left circumferential artery; LM, left main coronary artery; RCA, right coronary artery.

Supplementary Table S2. Time to composite clinical outcome in nitrate group and other types of vasodilator group

Time to event	Nitrates (n = 10)	Other types of vasodilator (n = 13)
Patient #1	40days	40days
Patient #2	47days	70 days
Patient #3	227days	96 days
Patient #4	230 days	119 days
Patient #5	241 days	171 days
Patient #6	269 days	295 days
Patient #7	330 days	327 days
Patient #8	333 days	331 days
Patient #9	340 days	359 days
Patient #10	357 days	366 days
Patient #11		369 days
Patient #12		380 days
Patient #13		380 days

Supplementary Table S3. One-year clinical event rate of patients with VA according to types of vasodilator in other types of vasodilator

	Nicorandil (n = 521)	Molsidomine (n = 177)	Trimetazidine (n = 111)	*p* value*
Composite events	12 (3.1)	1 (0.8)	0 (0.0)	0.118
ACS	9 (2.3)	0 (0.0)	0 (0.0)	0.094
Cardiac death	0 (0.0)	1 (0.6)	0 (0.0)	0.484
VT or VF	1 (0.3)	0 (0.0)	0 (0.0)	0.772
AV block	2 (0.5)	0 (0.0)	0 (0.0)	0.595
All-cause death	0 (0.0)	1 (0.8)	1 (1.2)	0.127
Readmission or emergency room visits due to angina	41 (10.5)	21 (17.4)	3 (3.7)	0.009

ACS, acute coronary syndrome; AV, atrioventricular; VA, vasospastic angina; VF, ventricular fibrillation; VT, ventricular tachycardia

* Chi-square test

Supplementary Table S4. Baseline characteristics after propensity matching

	All (n = 348)	Nitrate (n = 174)	Other types of vasodilator (n = 174)	*p* value
Age, years	52.1 ± 11.3	53.5 ± 11.1	52.8 ± 11.4	0.585
Male, n (%)	237 (68.1)	119 (68.4)	118 (67.8)	1.000
BMI, kg/m^2	24.7 ± 3.6	24.7 ± 3.8	24.8 ± 3.4	0.815
SBP, mmHg	126.5 ± 18.5	127.4 ± 19.6	125.6 ± 17.4	0.372
DBP, mmHg	77.7 ± 12.7	78.2 ± 13.8	77.2 ± 11.5	0.483
Previous CAD, n (%)	23 (6.6)	12 (6.9)	11 (6.3)	0.829
Diabetes mellitus, n (%)	22 (6.3)	14 (8.0)	8 (4.6)	0.186
Hypertension, n (%)	145 (41.7)	78 (44.8)	67 (68.5)	0.232
Dyslipidemia, n (%)	70 (20.1)	35 (20.1)	35 (20.1)	1.000
Alcohol drinking, n (%)	205 (58.9)	103 (59.2)	102 (58.6)	1.000
Current smoking, n (%)	138 (39.7)	67 (38.5)	71 (40.8)	0.661
Previous cardiovascular medication				
Antiplatelet, n (%)	49 (14.1)	26 (14.9)	23 (13.2)	0.644
Stain, n (%)				
CCB, n (%)	65 (18.7)	35 (20.1)	30 (17.2)	0.492
Clinical diagnosis before ergonovine				
Angina, n (%)	324 (93.1)	166 (65.4)	158 (90.8)	0.091
Myocardial infarction, n (%)	10 (2.9)	3 (1.7)	7 (4.0)	0.337
Cardiac arrest, n (%)	2 (0.6)	2 (1.1)	0 (0.0)	0.499
Syncope, n (%)	5 (1.4)	4 (2.3)	1 (0.6)	0.371
VT or VF, n (%)	1 (0.3)	1 (0.6)	0 (0.0)	1.000
AV block, n (%)	1 (0.3)	0 (0.0)	1 (0.6)	1.000

AV, atrioventricular; BMI, body mass index; CAD, coronary artery disease; CCB, calcium channel blocker; DBP, diastolic blood pressure; SBP, systolic blood pressure; VF, ventricular fibrillation; VT, ventricular tachycardia.

Supplementary Table S5. One-year clinical event rate of patients with good drug compliance

	All (n = 491)	Nitrate (n = 104)	Other types of vasodilator (n = 387)	*p* value
Composite events	10 (2.0)	4 (3.8)	6 (1.6)	0.231
ACS	7 (1.4)	2 (1.9)	5 (1.3)	0.643
Cardiac death	-	-	-	-
VT or VF	1 (0.2)	1 (1.0)	0 (0.0)	0.212
AV block	2 (0.4)	1 (1.0)	1 (0.3)	0.379
All-cause death	2 (0.4)	1 (1.0)	1 (0.3)	0.379

ACS, acute coronary syndrome; AV, atrioventricular; VF, ventricular fibrillation; VT, ventricular tachycardia.

Supplementary Table S6. One-year clinical event rate of patients with poor-compliance during 1 year

	All (n = 285)	Nitrate (n = 82)	Other types of vasodilator (n = 203)	*p* value
Composite events	13 (4.6)	6 (7.3)	7 (3.4)	0.156
ACS	10 (3.5)	6 (7.3)	4 (2.0)	0.036
Cardiac death	1 (0.4)	0 (0.0)	1 (0.5)	1.000
VT or VF	1 (0.4)	0 (0.0)	1 (0.5)	1.000
AV block	1 (0.4)	0 (0.0)	1 (0.5)	1.000
All-cause death	1 (0.4)	0 (.0)	1 (0.5)	1.000

ACS, acute coronary syndrome; AV, atrioventricular; VF, ventricular fibrillation; VT, ventricular tachycardia.
